# Long-term body mass trajectories and hypertension by sex among Chinese adults: a 24-year open cohort study

**DOI:** 10.1038/s41598-021-92319-4

**Published:** 2021-06-21

**Authors:** Ruru Liu, Baibing Mi, Yaling Zhao, Shaonong Dang, Hong Yan

**Affiliations:** 1 Xi’an Center for Disease Control and Prevention, Xi’an, 710054 Shaanxi China; 2 Department of Epidemiology and Biostatistics, School of Public Health, Xi’an Jiaotong University Health Science Center, No. 76, Yanta West Road, Xi’an, 710061 Shaanxi China

**Keywords:** Epidemiology, Cardiovascular diseases

## Abstract

Evidence was limited on trajectory of body mass index (BMI) through adulthood and its association with hypertension. We aimed to evaluate their association by sex in large**-**scale study. Data were obtained from the China Health and Nutrition Survey (CHNS) from 1991 to 2015. Latent class trajectory analysis (LCTA) was used to capture BMI change trajectories. Hazard risks (HRs) were estimated from Cox proportion hazard regression. Among 14,262 participants (mean age, 38.8; 47.8% men), 5138 hypertension occurred (2687 men and 2451 women) occurred during a mean follow**-**up 9.6 years. Four body mass trajectory groups were identified as BMI loss, stable, moderate and substantial gain. Appropriately half of participants (48.0%) followed 1 of the 2 BMI gain trajectories, where BMI increased at least 3 kg/m^2^ overtime. Compared with participants with stable BMI, those gaining BMI substantially had higher risk of hypertension by 65% (HR 1.65, 95% CI 1.45–1.86) in male and 83% (HR 1.83, 95% CI 1.58–2.12) in female. The HRs in BMI loss patterns were 0.74 (0.62–0.89) in men and 0.87 (0.75–1.00) in women. Our findings imply that majority of Chinese adults transited up to a higher BMI level during follow-up. Avoiding excessive weight gain and maintaining stable weight might be important for hypertension prevention.

## Introduction

Hypertension, an important public-health challenge worldwide, has strong association with the occurrence of all cardiovascular disease across a wide age range^1^, and holds the second most important global risk factor accounting for 9.6% of all disability-adjusted life-years^2^. The number of adult hypertension is predicted to be approximately 60% to a total of 1.56 billion (1.54–1.58 billion) in 2025, while its prevention and control are more challenging in low- and middle-income countries where health system are weak^3^. In Chinese adults, the hypertension prevalence almost sextupled from 5.1% in 1959 to 29.6% in 2014^4^, and might continue to rise in the next decade^5^. Therefore, it is of great importance to allocate and enhance prevention and management of hypertension.

Body mass index (BMI), the indicator for body fat mass recommended by World Health Organization, is the most frequently used diagnostic tool in the current classification system of overweight/obesity^6^. Numerous population studies across continents have demonstrated its utility as an estimate of risk with adverse health outcomes^7–9^. It is well-known that the potential causal association between BMI and hypertension was supported by long-term randomized controlled trials and prospective observational studies^10–12^. Moreover, evidence has been increasingly accumulated that BMI change is associated with additional health outcomes, compared to static weight status^13,14^. These studies implied that the BMI change trajectories might be curvilinear and varied across race, and suggested that higher hypertension might attribute to steeper increase in the slope of BMI^9,15–20^. These efforts, although useful, had several important limitations, including inaccurate calculation of BMI change by self-reported weight and height with a limited number of measurements, insufficient interpretation for gender difference, insufficient identification of within-person variation of physical activity or other lifestyle factors over time, and insufficient interpretation for the possible interaction with baseline weight status. Actually, most of these studies covered specific age groups with small samples, without estimating BMI–hypertension link over the life course. As yet, data has been spare regarding the association of BMI change trajectory with hypertension in Chinese adults, who are suffering from increasingly severe epidemic of overweight/obesity^21^. The only recent cohort study reported that sharp-increasing BMI trajectory group had a markedly higher risk of hypertension in Chinese health examinees, compared with the low-stable trajectory group^15^. However, this small-sample data was from routine health examinations in one single medical institution, which might limit its generalization to general population.

We tried to address these limitations by utilizing the data from China Health and Nutrition Survey (CHNS), a national representative open cohort study. In analysis, the gender-specific BMI change trajectory through adulthood was identified using latent class trajectory analysis (LCTA). We also attempted to outline the specific BMI change trajectory related to higher risk of hypertension in order to assist policymakers in developing the targeted intervention strategies.

## Results

### The characteristics of participants

During a mean follow-up 9.6 years, 5138 hypertension occurred. The prevalence of hypertension in men was 39.4%, higher than that in women (33.0%, *p* < 0.001). The average age and education years of individuals at baseline was 38.8 ± 14.2 and 7.4 ± 4.3 years. The majority of participants (63.0%) lived in rural areas. Over 30% of subjects were initial smoker or drinkers, with the average PA of 65.8 ± 95.9 MET-hours per week. There were significant disparity on most characteristics between men and women. Compared with women, men tended to be better educated, acquire higher income, be current smoker or drinker and consume more energy. There was also significant gender difference in SBP, DBP, BMI and hypertension prevalence (Supplementary Table S1).

### Long-term BMI change trajectory

As a result of group-based trajectory modeling analysis, the model with four trajectories had a better fit (lowest BIC) than that with other number of trajectories. The four potential trajectories were identified and characterized as BMI loss (a total average decrease of nearly 3 kg/m^2^), stable (change within 1 kg/m^2^), moderate gain (increase of nearly 3 kg/m^2^) and substantial gain (increase of nearly 6 kg/m^2^). The probability for each group was statistically significant (*p* < 0.01). The average posterior probability assignment ranged from 75.2 to 85.6%, and indicating a good discrimination of trajectory. (Supplementary text, Supplementary Tables S2 and S3). The proportions by class were 5.68%, 40.94%, 40.85%, 12.52% in men and 10.01%, 46.90%, 35.55%, 7.55% in women. A significant disparity was detected across gender as men were more likely to gain weight, but less like to lose weight, compared with women (χ^2^ = 227.64, *p* < 0.001).

**Figure 1:**
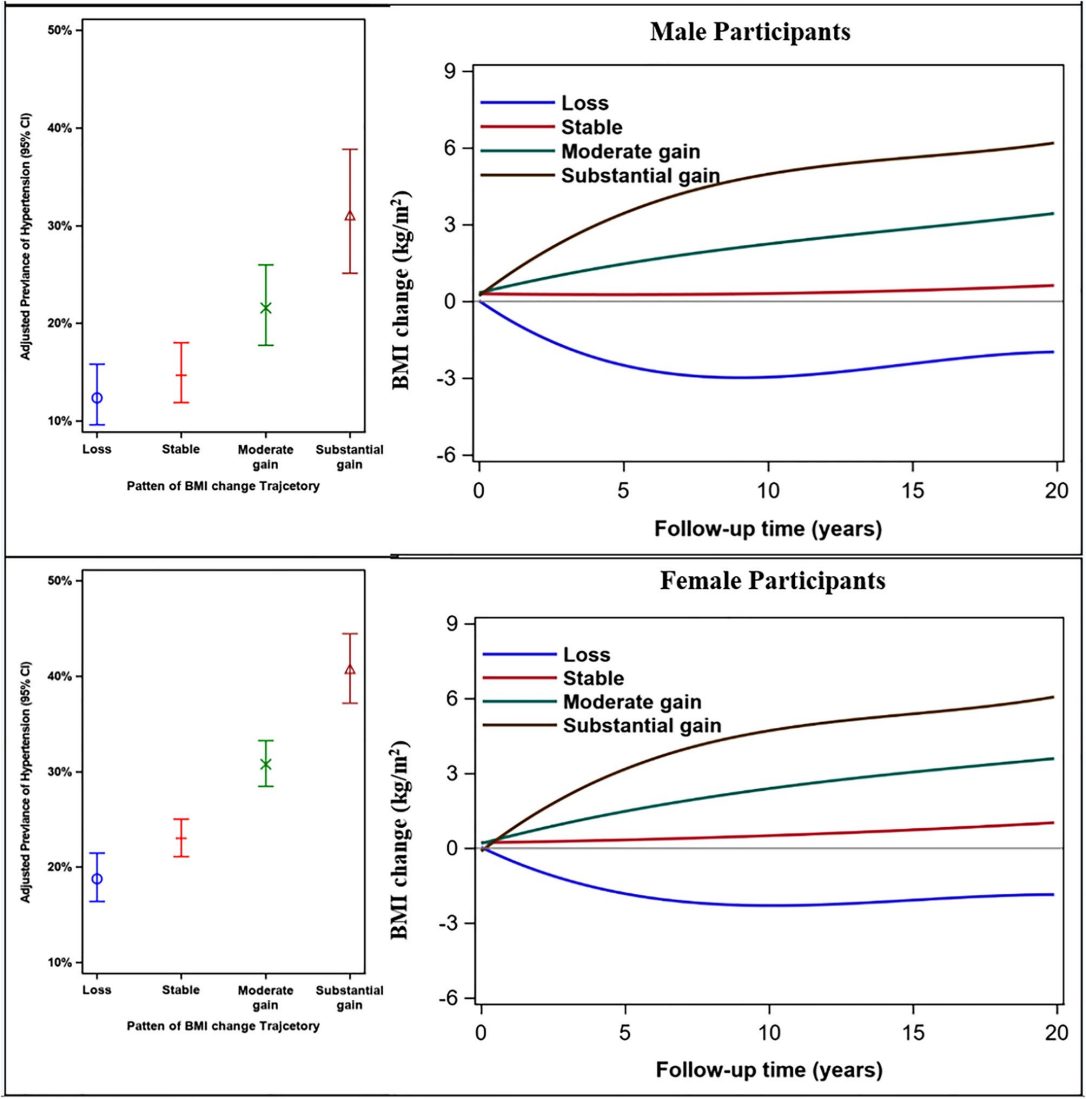
The probability of hypertension by BMI change trajectories across gender. Expected percentage (95% confidence interval) with elevated hypertension are presented from left to right corresponding to the relative level of BMI change in the color-matched trajectory. For ease of interpretation, BMI substantial gain trajectory are presented in brown, moderate gain in green, stable trajectory in red and BMI loss trajectory in blue. BMI indicates body mass index.

The trajectories of the four groups could be interpretable in context of the conventional BMI categories (Fig. 1). Starting from the flattest to the steepest trajectory classes, BMI stable trajectory represented the trajectory group with slight BMI change during the cohort period. In moderate gain trajectory in which the average BMI value increased by 3 kg/m^2^, and entered into overweight range. The remaining increase group (“substantial gain”) started out on a trajectory towards the greater end of obesity range, with the value increasing considerately. This trajectory covered 9.9% of participants who reached an average BMI of 26.5 kg/m^2^. The only decrease group (“loss”, 7.9% of total participants) saw the BMI values reduced from 25.5 to 22.3 kg/m^2^ for men and 25.4 to 22.6 kg/m^2^ for women during the follow-up period.

### Participant characteristics across BMI change trajectory

Table 1 shows distribution of demographics and lifestyle characteristics across BMI change trajectories by sex. Younger adults had higher odds of BMI gain relative to stable group. Percentage of current smoker varied significantly across the four trajectory groups in males but not in females. All physical measurement including SBP/DBP, BMI, weight and PA level were different among these pattern groups (all *p* < 0.001). Higher proportion of obese individuals at baseline followed BMI loss trajectory, compared with non-obese participants, regardless of gender.

**Table 1:** Characteristics among participants among 4 groups of BMI change trajectory by sex^a^.

Characteristics	Loss	Stable	Moderate gain	Substantial gain	Statistics^b^	*p*
**Male**						
No. participants	388	2795	2789	855		
Total person × years	3221	26,425	27,695	8453		
Follow-up duration (year)	8.3 (6.1)	9.5 (7.0)	9.9 (7.1)	9.9 (6.5)	7.49	< 0.001
Age (y)	41.8 (15.8)	40.4 (14.1)	37.0 (14.0)	32.8 (13.0)	79.47	< 0.001
Education year (y)	8.8 (4.2)	7.9 (4.0)	8.2 (3.6)	9.0 (3.4)	308.59	< 0.001
Rural, n (%)	214 (55)	1802 (64)	1824 (65)	553 (65)	15.70	0.001
Initial SBP (mmHg)	118.3 (10.9)	114.8 (11.1)	113.3 (10.8)	113.6 (11.0)	28.77	< 0.001
Initial DBP (mmHg)	76.4 (7.2)	74.8 (7.8)	73.8 (7.8)	74.0 (7.8)	18.23	< 0.001
Initial weight (kg)	71.0 (11.1)	61.9 (10.2)	59.3 (8.5)	59.5 (8.1)	193.13	< 0.001
Initial BMI (kg/m^2^)	25.5 (2.9)	22.3 (2.8)	21.2 (2.4)	21.0 (2.3)	370.40	< 0.001
Lean (< 18.5), n (%)	1 (0.3)	150 (5.4)	293 (11)	110 (13)	885.03	< 0.001
Normal (18.5–23.9), n (%)	129 (33)	1935 (69)	2152 (77)	657 (77)		
Overweight (24.0–27.9), n (%)	180 (46)	599 (21.0)	308 (11)	81 (9.5)		
Obesity (≥ 28.0), n (%)	78 (20)	111 (4.0)	36 (1.3)	7 (0.8)		
Current weight (kg)	62.6 (11.4)	61.5 (10.9)	65.6 (9.9)	74.7 (10.1)	356.39	< 0.001
Current BMI (kg/m^2^)	22.3 (3.3)	22.2 (3.1)	23.5 (3.0)	26.4 (2.9)	445.92	< 0.001
Lean (< 18.5), n (%)	49 (13)	268 (9.6)	106 (3.8)	3 (0.4)	1106.50	< 0.001
Normal (18.5–23.9), n (%)	224 (58)	1811 (65)	1499 (54)	170 (20)		
Overweight (24.0–27.9), n (%)	91 (23)	593 (21)	992 (36)	251 (29)		
Obesity (≥ 28.0), n (%)	24 (6.2)	123 (4.4)	192 (6.9)	431 (50)		
Current smoker, n (%)	206 (53)	1690 (61)	1672 (60)	456 (53)	21.42	< 0.001
Current drinker, n (%)	198 (51)	1571 (56)	1610 (58)	490 (57)	6.61	0.085
Current dietary energy (kcal)	2258.8 (2292.5)	2243.2 (792.8)	2305.5 (1128.1)	2358.4 (994.1)	3.02	0.028
Current physical activity (MET hours/week)	121.1 (123.9)	130.2 (133.0)	132.3 (143.5)	154.7 (157.5)	7.77	< 0.001
**Female**						
No. participants	744	3487	2643	561		
Total person × years	6393	32,098	27,245	5564		
Follow-up duration (year)	8.6 (6.4)	9.2 (7.0)	10.3 (7.1)	9.9 (6.8)	18.43	< 0.001
Age (y)	42.4 (14.5)	40.3 (14.5)	37.8 (13.2)	36.8 (13.9)	33.99	< 0.001
Education year (y)	6.5 (4.6)	6.7 (4.6)	6.6 (4.5)	6.7 (4.5)	0.45	0.717
Rural, n (%)	431 (58)	2113 (61)	1728 (65)	343 (61)	21.14	< 0.001
Initial SBP (mmHg)	114.7 (12.1)	110.5 (12.0)	109.1 (12.1)	109.5 (11.5)	44.07	< 0.001
Initial DBP (mmHg)	74.5 (8.0)	72.0 (8.3)	71.3 (8.4)	71.4 (8.1)	30.17	< 0.001
Initial weight (kg)	61.3 (9.2)	54.0 (8.2)	51.7 (7.6)	51.8 (7.3)	287.76	< 0.001
Initial BMI (kg/m^2^)	25.4 (3.2)	22.3 (2.8)	21.2 (2.6)	20.9 (2.5)	481.75	< 0.001
Lean (< 18.5), n (%)	9 (1.2)	208 (6.0)	364 (14.0)	91 (16.0)	1159.78	< 0.001
Normal (18.5–23.9), n (%)	263 (35.0)	2412 (69)	1888 (71.0)	399 (71.0)		
Overweight (24–27.9), n (%)	319 (43.0)	757 (22.0)	360 (14.0)	67 (12.0)		
Obesity (≥ 28.0), n (%)	153 (21.0)	110 (3.2)	31 (1.2)	4 (0.7)		
Current weight (kg)	55.2 (10.1)	54.2 (9.0)	57.8 (9.0)	64.9 (9.1)	254.69	< 0.001
Current BMI (kg/m^2^)	22.6 (3.8)	22.4 (3.1)	23.8 (3.2)	26.6 (3.2)	337.11	< 0.001
Lean (< 18.5), n (%)	98 (13.0)	326 (9.3)	97 (3.7)	3 (0.5)	871.97	< 0.001
Normal (18.5–23.9), n (%)	403 (54.0)	2183 (63.0)	1330 (50.0)	125 (22.0)		
Overweight (24–27.9), n (%)	165 (22.0)	818 (23.0)	941 (36.0)	243 (43.0)		
Obesity (≥ 28.0), n (%)	78 (10.0)	160 (4.6)	275 (10.0)	190 (34.0)		
Current smoker, n (%)	33 (4.4)	122 (3.5)	85 (3.2)	12 (2.1)	5.55	0.136
Current drinker, n (%)	55 (7.4)	287 (8.2)	202 (7.6)	40 (7.1)	1.45	0.695
Current dietary energy (kcal)	1915.1 (2202.2)	1891.0 (714.0)	1911.2 (642.5)	1948.8 (621.6)	0.72	0.542
Current physical activity (MET hours/week)	122.5 (107.1)	128.1 (120.4)	128.1 (130.2)	138.6 (123.2)	2.06	0.103

^a^Values in table are mean ± SD or N (percent); Missing data are handled in the analysis.

^b^F value for continues variable and Chi square for category variable.

### Association of BMI change trajectory with risk of hypertension

Table 2 shows the association of BMI change trajectory with hypertension. Both moderate and substantial gain patterns were associated with increased risk of hypertension, after adjusting for multiple potential risk factors, regardless of gender. Compared with stable trajectory, substantial weight gain was associated with increased hypertension by 65% (95% CI 1.45–1.86) in males and 83% (95% CI 1.58–2.12) in females. In fact, 38.1% of men and 29.9% of women in BMI stable trajectory were reported to be have hypertension, but the figures reached 42.5% for males and 39.9% for females in the substantial gain trajectory (Fig. 1). The BMI loss group was associated with lower risk of hypertension, the multivariate HRs were 0.74 (95% CI 0.62–0.89) in males and 0.87 (0.75–1.00) in females.

**Table 2:** Multivariate Cox regression analysis for the associations between BMI change trajectories and hypertension by sex.

BMI change trajectories	Case/N	Unadjusted model^b^		Adjusted model^c^	
		HR (95% CI)^a^	*p* value	HR (95% CI)^a^	*p* value
**Male**					
Loss	139/388	1.10 (0.92–1.31)	0.287	0.74 (0.62–0.89)	0.002
Stable	1064/2795	Ref.		Ref.	
Moderate gain	1121/2789	1.00 (0.92–1.09)	0.991	1.23 (1.13–1.34)	< 0.001
Substantial gain	363/855	1.08 (0.96–1.21)	0.224	1.65 (1.45–1.86)	< 0.001
**Female**					
Loss	259/744	1.28 (1.11–1.46)	< 0.001	0.87 (0.75–1.00)	0.053
Stable	1044/3487	Ref		Ref	
Moderate gain	924/2643	1.03 (0.94–1.12)	0.582	1.28 (1.17–1.40)	< 0.001
Substantial gain	224/561	1.24 (1.07–1.43)	0.003	1.83 (1.58–2.12)	< 0.001

^a^*HR* hazard ration, *CI* confidence interval.

^b^Undjusted model is crude model without any covariates.

^c^Adjusted model adjusted variables including age, survey wave, initial BMI, initial SBP/DBP, change of PA and dietary energy intake, change of smoking and drinking status.

**Figure 2:**
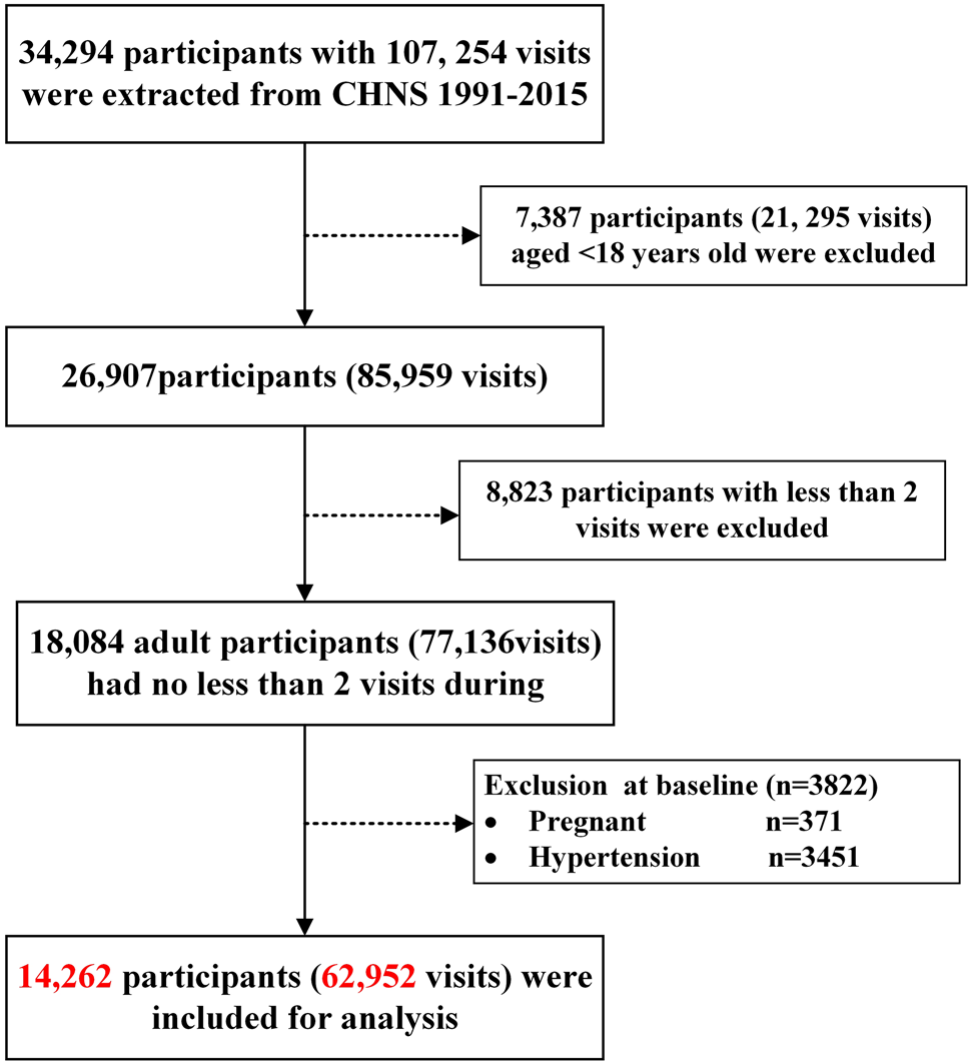
The flow diagram of participants in CHNS from 1991 to 2015.

Table 3 shows the joint association of BMI change trajectory and initial BMI status with hypertension by sex. The risk of hypertension generally increased in a gradual manner from moderate gain to substantial gain group in non-overweight participants. Compared with participants who were in BMI stable group, those women in substantial gain group and were non-overweight at baseline were at the highest risk (HR 1.86, 95% CI 1.58–2.19). The positive association weakened among initial overweight individuals and turned insignificance for females. It was notably that BMI loss trajectory witnessed 32% (HR 0.68, 95% CI 0.54–0.87) reduction in risk among overweight men at baseline, and the figure reduced to 27% with no statistical significance in overweight women. Meanwhile, among initial non-overweight adults, BMI loss was not associated with hypertension [HR 1.06 (0.83–1.34) in men and 0.83 (0.69–1.00) in women].

**Table 3:** Joint association of BMI change trajectories and BMI at baseline with risk of hypertension by sex.

BMI change trajectories	Male^b^		Female^b^	
	HR (95% CI)^a^	*p* value	HR (95% CI)^a^	*p* value
**Non-overweight**				
Loss	1.04 (0.78–1.37)	0.808	1.06 (0.83–1.34)	0.645
Stable	Ref.		Ref.	
Moderate gain	1.22 (1.11–1.35)	< 0.001	1.33 (1.19–1.47)	< 0.001
Substantial gain	1.64 (1.43–1.88)	< 0.001	1.86 (1.58–2.19)	< 0.001
**Overweight**				
Loss	0.68 (0.54–0.87)	0.002	0.83 (0.69–1.00)	0.052
Stable	Ref.		Ref.	
Moderate gain	1.24 (1.04–1.49)	0.019	1.13 (0.94–1.35)	0.188
Substantial gain	1.52 (1.11–2.09)	0.010	1.61 (1.14–2.29)	0.007

^a^*HR* hazard ration, *CI* confidence interval.

^b^The HRs were adjusted for age, survey wave, initial BMI, initial SBP/DBP, change of PA and dietary energy intake, change of smoking and drinking status.

### Subgroup analysis

We found no significant interaction in prespecified subgroup for hypertension risk for BMI change trajectories in males (*p* for interaction > 0.05; Supplementary Fig. S1). These different strata all provided similar results with our main findings. However, BMI at baseline was the main source of heterogeneity in females (*p* for interaction = 0.004). Moderate or substantial BMI gain was not associated with hypertension risk among participants who were obese at baseline (HR 0.75, 0.41–1.36; 1.41, 0.19–10.56). Additionally, BMI loss trajectory was related to decreased risk of hypertension by 23% (HR 0.77, 0.62–0.97) among initial overweight subjects but not among other population (Supplementary Fig. S2). Similar heterogeneity was observed in males, but the interaction weakened into insignificance (*p* for interaction = 0.118).

### Sensitivity analysis

Sensitivity analysis was conducted by excluding the participants with a history of chronic diseases and follow-up years ≤ 4 years, and the HRs did not significantly change in both men and women. Findings by multiple imputation techniques did not differ from those from the analytic samples, with the direction and magnitude of the association persisted. Detailed results was shown in Supplementary Table S4.

## Discussion

In this large-scale open cohort study Chinese adults, we identified 4 distinct BMI change trajectory using latent trajectory method. The majority of Chinese adults (53.8% men and 43.1% women) followed weight gain pattern and transited up to a higher BMI level during follow-up. Long-term weight gain was associated with increased risk of hypertension, compared with individuals with stable weight. The increased risk was more striking among women who were in substantial gain group and non-overweight at baseline. Although weight loss pattern was related with lower hypertension, the interpretation should be cautious due to the small sample size and great BMI variation of this group. Our study suggested avoiding excessive BMI gain and maintaining reasonable weight might be helpful to the prevention of hypertension. Further study was needed to confirm our finding and estimate long-term effect of weigh loss.

In current analysis, we identified 4 distinct, mutually exclusive BMI change groups with trajectory method by gender and examine the relative contributors of behaviors and characteristic to their trajectory profiles. This might be favorable for public health strategies targeted those with higher risk of weight gain across adulthood. During the 24-year period, appropriately half of participants (48.0%) of individuals were likely to gain weight significantly and transit up into overweight or obesity over time. It might be likely that these numbers would be greater for today’s adults, considering that today’s environment is even more obesogenic than that in 20 years ago, especially in Chinese^22^. Our study revealed that men were more likely to follow the higher BMI trajectory compared to women, as they tended to ignore weight management somewhat or they were more likely to be drinker, consistent with previous trajectory studies^9,18,23^. In addition, the substantial gain group tended to be younger adults. The evidence of substantial weight gain during just a few years might be a strong indicator that intensive weight management intervention is warrant^16,18^. Our results showed that smoking was associated with a decreased risk of weight change in men, which was consistent with previous studies that smoking was related to lower initial BMI and slower change rate over time^23,24^. Inconsistent with study in American and recent report of Chinese^9,15^, we did classify a trajectory of weight loss. Participants in this trajectory were elder and more likely to be women, and overweight or obese at baseline. We could not examine the possible reasons for the loss, limited by data and further study was needed to address this.

Our study indicated that long-term BMI gain was associated with increased risk of hypertension in both Chinese men and women, and the steeper BMI gain might place individuals at greater risk irrespective of initial BMI. This was consistent with the results reported in Canadians and Japanese cohort studies^16,25^. Of note, the hazard risk estimates appeared to be stronger in women than in men, consistent with results from two prospective US cohort studies^26^. Nevertheless, strict comparison between genders was difficult to conduct, due to the fact that trajectories were extracted by sex and sex-specific reference groups were adopted in analysis. We also observed a joint association between BMI change trajectories and initial BMI. Participants who were non-overweight and had substantial BMI gain were at higher risk of hypertension than those who did not have either of the conditions. This is consistent with the results reported in Framingham Heart Study offspring cohort that BMI variability, compared with stable BMI, was associated with 74% higher risks of having hypertension among non-obese participants^27^.

The mechanism for the associations of increased body weight with hypertension remains unclear. However, there were several plausible hypotheses. It was reported that blood volume expansion and renal sodium reabsorption were main features in the development of obesity–hypertension in both experimental models and humans^28^. Higher BMI was associated with adipose tissue dysfunction, characterized by increased infiltration by macrophages and marked changes in secretion of adipokines and free fatty acids, and enlarged hypertrophied adipocytes^29^. And oxidative stress, activation of the renin–angiotensin–aldosterone system and sympathetic overdrive, chronic vascular inflammation might be the important mechanisms involved^29^. However, it was unable to test theses hypothesis with available data in present study. Further studies were necessary to explore the precise reasons.

Another sobering finding of present study was that those individuals who successfully lost weight tended to be accompanied by lower hypertension risk, which was consistent with previous study in other population^12^. However, when we took the initial BMI status into account, the negative relation persisted among overweight participants but attenuated to insignificance among non-weight subjects. Whereas, we should be cautious to draw the conclusion of the beneficial effect of weight loss, as the robustness of results might be limited by the great BMI variation and small sample in this trajectory. Additionally, there might be reverse causation from pre-existing (such as undiagnosed cancer or other disease) that could result in weight loss, as there was a large number of elderly individuals. The inability to tackle this issue might result in an inaccurate or even spurious inverse association between weight change and health outcome^26^. In fact, results from National Health and Nutrition Examination Survey (NHANES) suggested that weight loss in middle to late adulthood was associated with increased risk of all-cause and heart disease mortality^30^. Further studies focusing on the long-term health effect of weight loss were warranted.

This is the largest longitudinal analysis of BMI change patterns and their interaction with initial BMI by gender to be conducted in a diverse sample of Chinese adults. Compared with traditional analysis, the LCTA approach we used had unique advantage to describe the developmental course and classify participant into distinct, mutually exclusive groups. The BMI and BP measures followed standardized protocols, avoiding self-report bias, particularly the underestimation in women who tended to underreport their weight and more at high BMI level^31^. Our findings might not be due to chance, as the association remained significant in subgroup and sensitivity analysis. Several limitations should be addressed. Firstly, residual confounding cannot be fully avoided, although we controlled detailed possible covariates. Secondly, we did not evaluate effects of active or latent diseases during the follow-up which might result in the weight change and bias the association of BMI change trajectories and hypertension. And we could not differentiate whether weight change was intentional or not, and additional information for the possible underlying reason for weight change might be valuable for better interpretation. Thirdly, the generalizability of main findings for those excluded participants or other races might be limited because of the significant disparity of characteristics between analytic and excluded samples. Fourthly, it might be difficult to assess weight circle when including participants with 3 time point or less, although the LCTA method was flexible to deal with different observation times between participants. However, our trajectory building model had good discriminant by and could tracked BMI change well across trajectories, which indicated that these trajectories could parsimoniously summarize the predominant features of BMI change pattern in our population. Additionally, the fact that we obtained similar results after excluding participants with follow-up period ≤ 4 years was reassuring. Lastly, our findings should be interpreted with caution due to the open cohort design. Actually, participants might enter and leave in any wave in current analysis. It was reported that trajectory approach would map BMI change pattern more favorably, if participants are enrolled at same time point and follow-up records come from the population without persons replaced^32^. Therefore, further studies are needed to evaluate our findings and explore the potential biological pathways between long-term weight gain and hypertension pathogenesis.

In conclusion, our findings underscore the necessity for future public health guidelines to assess long-term weight change trajectory in Chinese adults, as the majority of participants gained weight and transited up to a higher BMI level during follow-up. Furthermore, long-term BMI gain was associated with higher risk of hypertension, whereas, weight loss had contrary relation. As weight loss was less achievable (7.94% of total participants) and involved great BMI variation, our study indicated that prevention of weight gain might be more helpful. Taken together, our study suggested avoiding excessive weight gain and maintaining stable weight might be important for hypertension prevention. Further studies was warranted to unravel the mechanism underlying the association between BMI change and hypertension and to consider the long-term health consequence of weight loss.

## Methods

### Study population

The data used in present study was from the CHNS, an ongoing open cohort, which examined a series of economic, sociological, demographic and health questions. The open database, study materials and acknowledgement is available at the website (http://www.cpc.unc.edu/projects/china). A multistage random cluster process was used to draw a sample of about 7200 households with over 30,000 individuals in 15 provinces. CHNS rounds have been achieved in 1989, 1991, 1993, 1997, 2000, 2004, 2006, 2009, 2011 and 2015. Detailed description of study design and procedures was available in the published cohort profile^33^. All participants signed the informed consent and this study was approved by the institutional review committees of the National Institute of Nutrition and Food Safety, Chinese Center for Disease Control and Prevention, the University of North Carolina at Chapel Hill, and the China-Japan Friendship Hospital, Ministry of Health. All methods were carried out in accordance with relevant guidelines and regulations.

In present study, the first survey wave was set as 1991 when detailed information on lifestyle factors and diet information was collected. The end of follow-up was 2015 and analysis data was based on 9 waves of CHNS from 1991 to 2015. The flow diagram of participants involved in current study was showed in Fig. 2. A total of 34,292 participants with 107,254 visits were extracted from the original surveys. However, we excluded 7384 participants who aged < 18 years old, 8823 participants with less than 2 visits during the follow-up period. We further exclude 3822 participants including pregnant women or hypertension patients at baseline. Finally, 14,262 (6827 males and 7435 females) remained for the final analysis. A comparison of main characteristics of excluded and analytic sample was shown in Supplementary Table S5. Compared with participants included in this study, those excluded were younger, more likely to be men, and more likely to be better educated and higher PA level, tended to live in rural areas and consume less total dietary energy (all *P* < 0.05). Excluded participants have more favorable measurement of BP, BMI (all *P* < 0.001).

We conducted a post hoc analysis to calculate the power to detect a significant association of BMI change with hypertension, based on the existing sample size in our study and a moderate effect size. The hypertension prevalence in Chinese adult was 29.6%^4^, and the hazard ratio (HR) of ‘substantial gain’ pattern assumption was 1.2. The results indicated that current sample size was large enough to detect the assumed effect size, with estimated statistical power > 90%, with type I error of 0.05. The calculation was performed with an online power calculation tool (https://powerandsamplesize.com/Calculators/Test-Time-To-Event-Data/Cox-PH-Equivalence).

### Definition of follow-up in the study

Individuals included in current analysis were followed prospectively from their first visit in the CHNS. The study was an open cohort, as participants might enter and leave in any wave, and those lost to follow-up in one wave were still likely to enter the survey in other waves. Therefore, the duration of follow-up duration was defined as period from the first to latest visit, attended with information on mortality, end of follow-up, or loss to follow-up.

### Physical measurement and hypertension assessment

Height and weight at every wave were measured by trained healthcare staff using a reference protocol recommended by the WHO^34^. Weight was measured to the nearest 0.1 kg with participants in lightweight clothing. Height was measured to 0.1 cm with participants being barefoot. Further detail about CHNS has been reported elsewhere^33^. BMI was calculated as weight (kg)/square of height (m^2^).

Participants were asked to rest 10 min in the seated position prior to blood pressure measurement. The arterial blood pressure was measured using standard mercury sphygmomanometers, with 30-s intervals between cuff inflations. SBP and DBP were recorded as phase I and V Korotkoff sounds. The average of three measurements was used for analysis. According to the Seventh Report of the Joint National Committee on Detection, Evaluation, and Treatment of High Blood Pressure (JNC 7), hypertension was ascertained if SBP ≥ 140 mmHg and/or a DBP ≥ 90 mmHg, or the use of antihypertensive medications, or a physician diagnosis of hypertension^35^.

### Covariates ascertainment

Evidence from large-scale prospective studies suggested that population with stable weight tended to have higher socioeconomic position, healthier lifestyle factors and consumed a healthier diet than those in weight change groups^26,30^. According to these studies and a priori knowledge about our data (results from the association between weight change pattern and risk of type 2 diabetes)^36^, a set of covariates were considered as potential confounders.

Demographic information including gender, age, education, residence and income level were collected using standard interview questionnaire. Education level was represented as total years of schooling, and was classified into never, no more than 6 years, 6–8 years, 9–11 years and no less than 12 years. Residence was categorized as urban and rural. Income was measured in RMB and collected from total household.

Participants were asked to report their current status of smoking in each wave, and were assigned to never or current smoker according to their answer to the question “Have you ever smoked cigarettes (including hand-rolled or device-rolled)”^36,37^. Alcohol consumption was classified into never or current, similar to smoking. Physical activity (PA) was measured by a semi-quantitative questionnaire, containing items of occupational, domestic, travel, and leisure in this study. PA intensity score, indicated by metabolic equivalent (MET) score, was calculated by multiplying frequency and duration of the activity converted to per week^38,39^.

The total energy intake was estimated by considering both the household and individual levels^40^. Household food consumption was estimated by conducting a detailed examination of changes in inventory for 3 consecutive days in combination with a weighing technique. Individual dietary information was asked to record each type and weight of food they consumed during 3 consecutive days^41^.

Amongst these variables, PA, smoking and drinking status and energy intake were predisposed as time-variant variables in large-scale cohort studies^36,42^. Therefore, in current analysis, the participant were assigned into 4 categories according to their changes smoking and alcohol consumption during follow-up period: (1) never to never (never smoking/drinking at baseline or the last survey wave), (2) never to current (begin smoking/drinking), (3) current to former (quit smoking/drinking), (4) current to current (keep smoking/drinking). PA and energy intake change level were classified as increase or decrease, by calculating their values at baseline and current.

To avoid potential over-adjustment, we used directed acyclic graph (DAG) (Supplementary Fig. S3) to minimize number of variables in multivariate model^43,44^. Finally, 9 variables including age (continuous), survey wave (categorical survey year), initial BMI (continuous), initial SBP/DBP continuous (continuous), change of PA and dietary energy intake (decrease or increase), change of smoking and drinking status (never to never, never to current, current to never or current to current) were included.

### Statistical analysis

Some prior studies including Nurses’ Health and Health Professionals follow-up study in US, the Health Survey in England (1992–2010), have observed a gender disparity in the association between weight change trajectories and health outcomes^26,45^. In current analysis, we examine the interaction by creating a cross product of sex and BMI change trajectories. In the regression model, this variable was significantly associated with event (Wald χ^2^ = 47.980, *P* < 0.001). Likelihood ratio test comparing with and without this variable conforms this result with significance. Thus, we stratify the sample by gender to better understand this relationship. We calculated the follow-up person years from the date of returning the participants first enrolled to the date of hypertension or the end of cohort, whichever came first. Demographic and health-related variable were summarized by gender, with number and percentage for categorical variables and mean and S.D. for continuous variables. Gender-specific baseline characteristics were compared by Chi-square test or *t* test.

We used LCTA method with the TRAJ procedure^12,46^, to describe BMI change trajectories and then assess their associations with hypertension. We modeled BMI change between each wave as BMI_current_ minus BMI_baseline_ over time. We repeated trajectory analysis with changing group number between 2–5, and both linear, quadratic and cubic of models were performed. According to framework to construct latent class trajectory modeling^32^, we considered several factors to determine the optimal model and number of trajectory groups, including a priori knowledge^9^, the Bayesian Information Criterion (BIC), significance of polynomial terms, value of average posterior probability (entropy) and of group membership probability, leaning towards parsimony in number of trajectory groups^47^. For each trajectory, we aimed for groups with membership probabilities of at least 5%^9,47^. Within each trajectory, the value of mean posterior probability of membership was ascertained; as the value of ≥ 75.0% indicated adequate internal reliability^48^. Each participant was assigned exclusively to the trajectory group with the highest posterior probability (maximum probability assignment rule). Trajectory membership was used as an indicator variable in present analyses.

We used Cox proportional hazards regression to calculate the unadjusted and adjusted HRs and corresponding 95% confidence intervals (CIs) of each BMI change trajectory, with stable group as reference. The adjusted covariates included the 9 variables defined by DAG. We examined the proportional hazards assumption by creating interaction terms of follow-up time and main exposure variables including BMI change patterns. Likelihood ratio tests comparing including and excluding these variable were not significant, suggesting no violations. We also evaluated this assumption by visual inspection of log–log plots, which were consistent with proportional hazards. We completed the main analysis by assuming that variables were assumed to be missing completely at random (MCAR), consistent with pervious study^36^.

To evaluate the possible joint association of initial BMI and BMI change on hypertension risk over time, we divided the participants into 2 groups by gender according to weight status at baseline (overweight and non-overweight), and repeated Cox proportional hazard regression with BMI stable trajectory as reference. Furthermore, We performed stratified analyses defined a priori by BMI at baseline (lean, normal, overweight and obesity), residence (urban or rural), age at enrollment (≤ 40 years old, 41–60 years and > 60 years), change of drinking status (never–never, never–current, drinker–quit and drinker–drinker), change of smoking status (never–never, never–current, smoker–quit and smoker–smoker), change of PA and dietary energy intake (decrease or increase). For each of these variables, we tested for potential effect modification by using likelihood ratio tests for interactions.

A series of sensitivity analysis was conducted to test the robustness of the results. Firstly, we used the subsample with excluding participants developed diabetes, myocardial infarction, stroke or cancer, to minimize the possible reverse causation caused by these disease. Secondly, we excluded individuals who had follow-up of no than 4 years during the follow-up, to test applicability of favorable models and its association with hypertension across gender. Thirdly, the multiple imputation by the chained equations models was used to deal with missing data. We used BMI change trajectories, hypertension and other demographics, anthropometry and health behavior variables in multivariate models as predicators. Linear regression models for continuous variables and discriminant functions for categorical variables were used to impute values. We generated 20 imputed data sets, as the total missing across these variables was lower than 15%. The multiple Cox regression analyses were repeated in each of the augmented data sets, parameter estimates were means of the 20 sets, and their SEs were calculated by the Rubin method. All analyses were conducted with SAS 9.4 (SAS Institute, Cary, NC). Two-sided tests were used and *p* < 0.05 was considered statistically significant.

## Supplementary Information

Supplementary Information.

## Data Availability

The data was obtained from CHNS, an open cohort study. Original database, study materials and acknowledgement is available at the website (http://www.cpc.unc.edu/projects/china). The analytic datasets are available from the corresponding author on reasonable request.
