# Supplementary material for: Long-term body mass trajectories and hypertension by sex among Chinese adults: a 24-year open cohort study
Source: Sci Rep. 2021 Jun 21;11:12915. doi: 10.1038/s41598-021-92319-4 (PMC8217242; doi:10.1038/s41598-021-92319-4)

Long-term body mass trajectories and hypertension by sex among Chinese adults: a 24-y open cohort study

Short title: Trajectory of body mass and hypertension

Authors names and affiliations:

Ruru Liu, PhD candidate† ‡, Baibing Mi, PhD ‡, Yaling Zhao, PhD‡, Shaonong Dang, PhD‡, Hong Yan, PhD‡

Ruru Liu and Baibing Mi contributed equally to this work.

† Xi'an Center for Disease Control and Prevention, Xi'an, Shaanxi Province, 710054, China.

‡ Department of Epidemiology and Biostatistics, School of Public Health, Xi'an Jiaotong University Health Science Center, Xi'an, Shaanxi Province, 710061, China

Supplementary Table S1 Characteristics of the participants including in the final analysis ‡

Characteristics	Total	Male	Female	Statistics	*P*
No. Participants	14262	6827	7435		
Total Person*years	137094	65794	71300		
Follow-up duration (year)	9.6±7.0	9.6 ±6.9	9.6±7.0	0.17	0.684
No. Hypertension, n (%)	5138 (36.0)	2687 (39.4)	2451 (33.0)	63.11	<0.001
Age (y)	38.8 ±14.2	38.2 ±14.3	39.3 ±14.1	24.27	<0.001
Education year (y)	7.4 ±4.3	8.2±3.8	6.7±4.6	432.32	<0.001
Rural, n (%)	9008 (63)	4393 (64)	4615 (62)	7.92	0.005
Height(cm)	161.1 ±8.4	166.8 ±6.6	155.8 ±6.2	10540.50	<0.001
Initial BMI(kg/m^2)	22.0 ±2.9	21.9 ±2.8	22.1 ±3.0	16.94	<0.001
Lean (<18.5) , n(%)	1226 (8.6)	554 (8.1)	672 (9.0)		
Normal (18.5-23.9) , n(%)	9835 (69)	4873 (71)	4962 (67)	36.54	<0.001
Overweight (24-27.9) , n(%)	2671 (19.0)	1168 (17)	1503 (20)		
Obesity (≥28.0) , n(%)	530 (3.7)	232 (3.4)	298 (4.0)		
Initial weight(kg)	57.3 ±9.8	61.1 (9.7)	53.7 (8.5)	2310.33	<0.001
Initial smoker, n(%)	4706(33.0)	4401 (65)	305 (4.1)	5864.68	<0.001
Initial drinker, n(%)	5169 (36)	4262 (63)	907 (12)	3877.24	<0.001
Initial SBP(mmHg)	112.2 ±11.8	114.2 ±11.0	110.3 ±12.1	405.53	<0.001
Initial DBP(mmHg)	73.1 ±8.2	74.4 ±7.8	72.0 ±8.4	316.54	<0.001

Initial Physical Activities (MET-hours/week)	65.8 ±95.9	49.0 ±96.6	81.2 ±92.4	393.05	<0.001
Initial dietary energy (kcal)	2406.9 ±948.0	2615.2 ±840.0	2218.1 ±999.4	636.57	<0.001
Current BMI(kg/m^2)	23.2 ±3.4	23.3 ±3.3	23.2 ±3.4	0.14	0.712
Lean (<18.5), n(%)	950 (6.7)	426 (6.2)	524 (7.0)		
Normal (18.5-23.9), n(%)	7745 (54.0)	3704 (54.0)	4041 (54.0)	9.59	0.022
Overweight (24-27.9), n(%)	4274 (30.0)	2107 (31.0)	2167 (29.0)		
Obesity (≥28.0), n(%)	1293 (9.1)	590 (8.6)	703 (9.5)		
Current weight(kg)	60.5 (11.2)	64.9 (11.2)	56.4 (9.6)	2364.02	<0.001
Current smoker, n(%)	4276 (30.0)	4024 (59.0)	252 (3.4)	5243.47	<0.001
Current drinker, n(%)	4453 (31.0)	3869 (57.0)	584 (7.9)	4279.84	<0.001
Current SBP(mmHg)	123.5 ±17.6	125.1 ±16.8	121.9 ±18.2	117.24	<0.001
Current DBP(mmHg)	79.0 ±10.6	80.6 ±10.5	77.6 ±10.4	305.38	<0.001
Current physical activities (MET hours/week)	131.6 ±131.6	133.7 ±140.7	129.5 ±123.2	3.51	0.061
Current dietary energy (kcal)	2086.2 ±1038.9	2283.9 ±1095.1	1904.9 ±949.0	488.05	<0.001

‡ Values in table are mean ± SD or N (Percent); Missing data are handled in the analysis; BMI, body mass index; SBP, systolic blood pressure; DBP, diastolic blood pressure

Supplementary Text. The model fitting procedure of BMI change trajectory and goodness of model fitting

Using a maximum-likelihood approach, LCTA estimated multiple regression models simultaneously and calculated from the model parameters the probability of each participants belonging to each trajectory group. The model fitting procedure of BMI change trajectory with Proc Traj is listed as follows:

Step 1. Decide on the optimal number of groups using substantive knowledge. Based on previous researches (1, 2) and our substantive knowledge about the long-term change of BMI characteristics, we decided to fit two- to five-group models of BMI change trajectories.

Step 2. Fit number of groups to data. To determine the optimal number of trajectory groups included in the model, we compared Bayesian Information Criteria (BIC) between models with different groups. A smaller BIC value indicated a better fit (3), but as the number of groups was increased across models, the additional reduction of BIC became smaller (**Supplementary Table S3**). Following the existing guidelines (4, 5), we chose the model when additional groups failed to increase BIC by at least half of the BIC in the previous model and the value of group membership probability ≥5%.

Step 3. Select the shape of the pattern of change for each group over time. After the number of groups was selected, we determined further the shape of each trajectory group using a stepwise approach in establishing polynomial order, with all groups initially set to cubic order. As the order of each trajectory was set to quadratic, linear, and intercept respectively, we compared the changes in BIC and the significance of parameters across models. Based on the substantive knowledge and statistical inference, the cubic trajectory pattern with four trajectory groups was regarded as the best fitting for the data.

Once a model was selected, we examined the posterior probabilities for each trajectory group to ensure all groups provided evidence for adequate model fitting. A general rule required an acceptable model to have a minimum average posterior probability of 0.70 for all trajectories (4). In this study, average posterior probabilities for all trajectories were at least 0.75 (**Supplementary Table S4**)

References

1. Ye M, Robson PJ, Eurich DT, Vena JE, Xu JY, Johnson JA. Changes in body mass index and

incidence of diabetes: A longitudinal study of Alberta's Tomorrow Project Cohort. Preventive medicine. 2018;106:157-63.

2. Wang M, Yi Y, Roebothan B, Colbourne J, Maddalena V, Wang PP, et al. Body Mass Index Trajectories among Middle-Aged and Elderly Canadians and Associated Health Outcomes. J Environ Public Health. 2016;2016:7014857.

3. Nagin DS. Analyzing developmental trajectories: a semiparametric, group-based approach. Psychological methods. 1999;4(2):139.

4. Nagin DS, NAGIN D. Group-based modeling of development: Harvard University Press; 2005.

5. Jones BL, Nagin DS, Roeder K. A SAS procedure based on mixture models for estimating developmental trajectories. Sociological methods & research. 2001;29(3):374-93.

Supplementary Table S2. Tabulated Bayesian Information Criterion(BIC) for all participants

Number of Groups	BIC	Group membership probability (%)				
		**Group 1**	**Group 2**	**Group 3**	**Group 4**	**Group 5**
2	-121977.99	58.70	41.30			
3	-117907.51	26.93	54.28	18.78		
**4**	**-115720.08**	**9.19**	**41.47**	**38.09**	**11.25**	
5	-114448.35	4.44	39.29	26.46	22.91	6.90

Supplementary Table S3. Parameters estimated for BMI trajectory change pattern ‡

	BMI trajectory change pattern	**Intercept term**	**Linear term**	**Quadratic term**	**Cubic term**	**Group membership probability (%)**	**Average posterior probability (%)**
All participants	Loss	0.069	-0.651	0.051	1.0×10-3	7.94	84.6
	Stable	0.263	0.0293	-0.002	1.00×10-4	44.0	75.2
	Moderate gain	0.278	0.287	-0.009	1.90×10-4	38.1	75.5
	Substantial gain	0.070	0.882	-0.051	0.001	9.93	85.6
Male	Loss	0.027	-0.780	0.062	1.0×10-3	5.68	75.8
	Stable	0.303	-0.015	0.002	-	40.94	81.1
	Moderate gain	0.339	0.273	-0.010	2.3×10-4	40.85	79.3
	Substantial gain	0.224	0.866	-0.050	1.1×10-3	12.52	87.4
Female	Loss	0.039	-0.556	0.041	9.2×10-4	10.01	84.1
	Stable	0.229	0.016	0.001	-	46.90	76.7
	Moderate gain	0.195	0.305	-0.01	1.8×10-4	35.55	79.8
	Substantial gain	-0.117	0.894	-0.053	0.001	7.55	80.5

‡ Parameter estimate presented the shape of each pattern of trajectory over time. Intercept term interpreted as the expected level of change of BMI in kg/m^2 at the first year of follow-up. Linear term interpreted as the linear slope of change of BMI by follow-up year. Quadratic term interpreted as the quadratic slope and cubic term interpreted as the cubic slope.

Supplementary Table S4. Sensitivity analysis of the associations between different patterns of BMI trajectories and the risk of hypertension ‡

BMI change trajectories	Exclude follow-up ≤4 yrs		Exclude chronic diseases		Multiple Imputation	
	HR(95% CI) †	*P* value	HR(95% CI) †	*P* value	HR(95% CI) †	*P* value
Male						
Loss	0.78 (0.63-0.96)	0.018	0.69(0.56-0.85)	0.001	0.72(0.61-0.85)	0.001
Stable	Ref.		Ref.		Ref.	
Moderate gain	1.26 (1.14-1.39)	<0.001	1.22(1.12-1.34)	<0.001	1.21(1.12-1.32)	<0.001
Substantial gain	1.79 (1.56-2.06)	<0.001	1.65(1.45-1.88)	<0.001	1.64(1.44-1.87)	<0.001
Female						
Loss	0.82 (0.70-0.98)	0.025	0.92(0.79-1.08)	0.327	0.85(0.73-1.00)	0.046
Stable	Ref.		Ref.		Ref.	
Moderate gain	1.30 (1.18-1.44)	<0.001	1.29(1.17-1.42)	<0.001	1.28(1.17-1.40)	<0.001
Substantial gain	1.86 (1.58-2.20)	<0.001	1.93(1.65-2.26)	<0.001	1.86(1.62-2.14)	<0.001

‡ all models adjusted variables including age at baseline, survey wave, BMI at baseline, initial SBP and DBP, current smoking and alcohol consumption, current physical activity and dietary energy intake.

† HR, hazard ration. CI, confidence interval

Supplementary Table S5 Characteristics of participants in analytic sample and excluded samples †

Characteristics	Analytic sample	Excluded sample	Statistics*	*P*
No. Participants	14262	18232		
Male(%)	6827(47.9)	8930(48.9)	3.96	0.047
Age (y)	38.8 ±14.2	22.7±19.1	88.11	<0.001
Education year (y)	7.4±4.3	7.6±4.4	4.11	<0.002
Rural, n(%)	9008 (63.0)	10939(90.0)	33.75	<0.001
Initial weight(kg)	57.3±9.8	42.3±21.7	76.66	<0.002
Initial BMI (kg/m^2)	22.0 ±2.9	19.9±5.0	44.63	<0.001
Initial SBP(mmHg)	112.2 ±11.8	110.7±20.1	7.91	<0.001
Initial DBP(mmHg)	73.1 ±8.2	71.2±20.1	10.06	<0.001
Initial smoker, n(%)	4706 (33.0)	4193(23.0)	402.30	<0.001
Initial drinker, n(%)	5169 (36.0)	4011(21.9)	800.91	<0.001
Physical Activities(MET-hours/week)	65.8±95.9	87.1±102.5	19.14	<0.001
Dietary Total Energy(kcal)	2086.2 ±1038.9	1759.3±1422.0	23.06	<0.001

† Values in table are mean ±SD or N (Percent); Missing data are handled in the analysis; BMI, body mass index; SBP, systolic blood pressure; DBP, diastolic blood pressure

***** t test for continuous variable and Chi square for category variable.

Supplementary Fig.S1. **Subgroup analysis for the associations between different patterns of BMI trajectories and the risk of hypertension in males.**

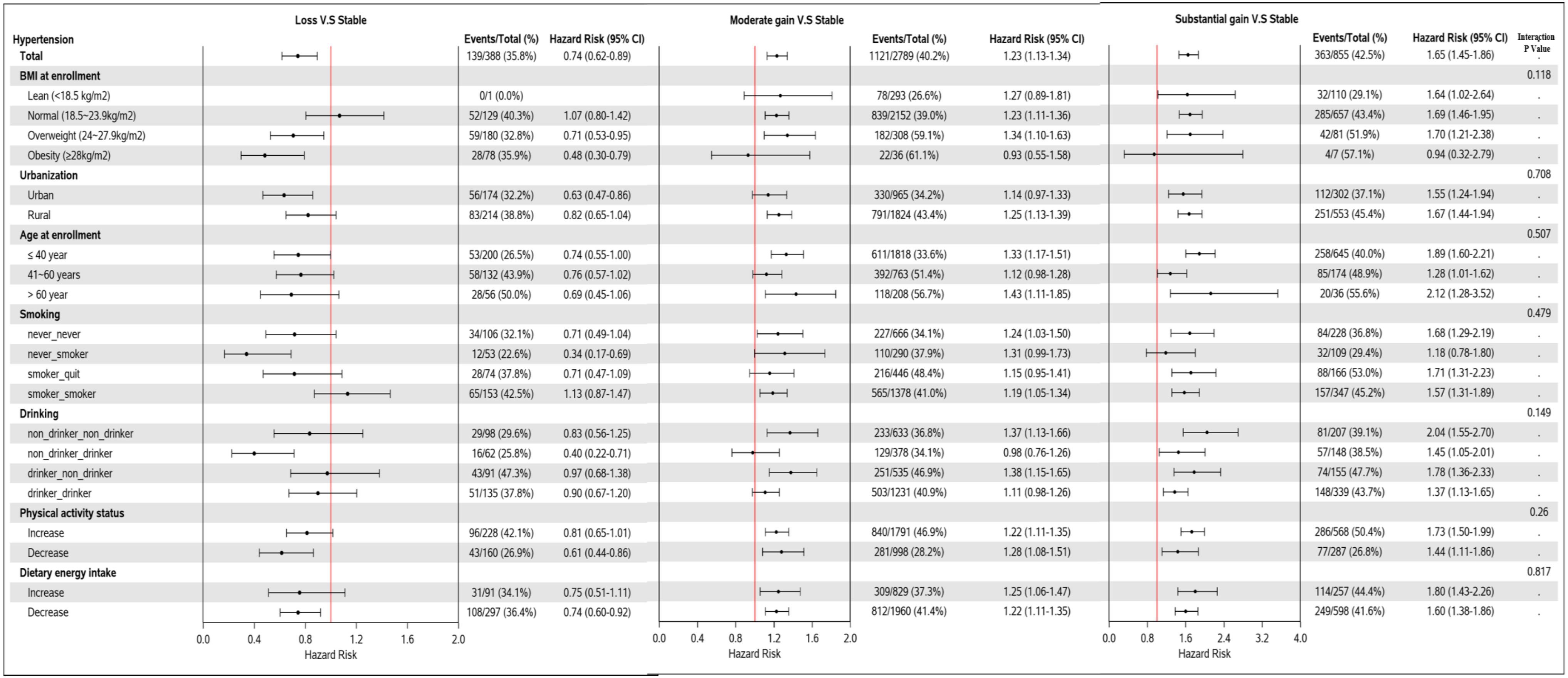

Hypertension	Loss V.S Stable Events/Total (%)	Loss V.S Stable Hazard Risk (95% CI)	Moderate gain V.S Stable Events/Total (%)	Moderate gain V.S Stable Hazard Risk (95% CI)	Substantial gain V.S Stable Events/Total (%)	Substantial gain V.S Stable Hazard Risk (95% CI)	Interaction P Value
**Total**	139/388 (35.8%)	0.74 (0.62-0.89)	1121/2789 (40.2%)	1.23 (1.13-1.34)	363/855 (42.5%)	1.65 (1.45-1.86)	.
**BMI at enrollment**							0.118
Lean (<18.5 kg/m2)	0/1 (0.0%)		78/293 (26.6%)	1.27 (0.89-1.81)	32/110 (29.1%)	1.64 (1.02-2.64)	.
Normal (18.5~23.9kg/m2)	52/129 (40.3%)	1.07 (0.80-1.42)	839/2152 (39.0%)	1.23 (1.11-1.36)	285/657 (43.4%)	1.69 (1.46-1.95)	.
Overweight (24~27.9kg/m2)	59/180 (32.8%)	0.71 (0.53-0.95)	182/308 (59.1%)	1.34 (1.10-1.63)	42/81 (51.9%)	1.70 (1.21-2.38)	.
Obesity (≥28kg/m2)	28/78 (35.9%)	0.48 (0.30-0.79)	22/36 (61.1%)	0.93 (0.55-1.58)	4/7 (57.1%)	0.94 (0.32-2.79)	.
**Urbanization**							0.708
Urban	56/174 (32.2%)	0.63 (0.47-0.86)	330/965 (34.2%)	1.14 (0.97-1.33)	112/302 (37.1%)	1.55 (1.24-1.94)	.
Rural	83/214 (38.8%)	0.82 (0.65-1.04)	791/1824 (43.4%)	1.25 (1.13-1.39)	251/553 (45.4%)	1.67 (1.44-1.94)	.
**Age at enrollment**							0.507
≤ 40 year	53/200 (26.5%)	0.74 (0.55-1.00)	611/1818 (33.6%)	1.33 (1.17-1.51)	258/645 (40.0%)	1.89 (1.60-2.21)	.
41~60 years	58/132 (43.9%)	0.76 (0.57-1.02)	392/763 (51.4%)	1.12 (0.98-1.28)	85/174 (48.9%)	1.28 (1.01-1.62)	.
> 60 year	28/56 (50.0%)	0.69 (0.45-1.06)	118/208 (56.7%)	1.43 (1.11-1.85)	20/36 (55.6%)	2.12 (1.28-3.52)	.
**Smoking**							0.479
never_never	34/106 (32.1%)	0.71 (0.49-1.04)	227/666 (34.1%)	1.24 (1.03-1.50)	84/228 (36.8%)	1.68 (1.29-2.19)	.
never_smoker	12/53 (22.6%)	0.34 (0.17-0.69)	110/290 (37.9%)	1.31 (0.99-1.73)	32/109 (29.4%)	1.18 (0.78-1.80)	.
smoker_quit	28/74 (37.8%)	0.71 (0.47-1.09)	216/446 (48.4%)	1.15 (0.95-1.41)	88/166 (53.0%)	1.71 (1.31-2.23)	.
smoker_smoker	65/153 (42.5%)	1.13 (0.87-1.47)	565/1378 (41.0%)	1.19 (1.05-1.34)	157/347 (45.2%)	1.57 (1.31-1.89)	.
**Drinking**							0.149
non_drinker_non_drinker	29/98 (29.6%)	0.83 (0.56-1.25)	233/633 (36.8%)	1.37 (1.13-1.66)	81/207 (39.1%)	2.04 (1.55-2.70)	.
non_drinker_drinker	16/62 (25.8%)	0.40 (0.22-0.71)	129/378 (34.1%)	0.98 (0.76-1.26)	57/148 (38.5%)	1.45 (1.05-2.01)	.
drinker_non_drinker	43/91 (47.3%)	0.97 (0.68-1.38)	251/535 (46.9%)	1.38 (1.15-1.65)	74/155 (47.7%)	1.78 (1.36-2.33)	.
drinker_drinker	51/135 (37.8%)	0.90 (0.67-1.20)	503/1231 (40.9%)	1.11 (0.98-1.26)	148/339 (43.7%)	1.37 (1.13-1.65)	.
**Physical activity status**							0.26
Increase	96/228 (42.1%)	0.81 (0.65-1.01)	840/1791 (46.9%)	1.22 (1.11-1.35)	286/568 (50.4%)	1.73 (1.50-1.99)	.
Decrease	43/160 (26.9%)	0.61 (0.44-0.86)	281/998 (28.2%)	1.28 (1.08-1.51)	77/287 (26.8%)	1.44 (1.11-1.86)	.
**Dietary energy intake**							0.817
Increase	31/91 (34.1%)	0.75 (0.51-1.11)	309/829 (37.3%)	1.25 (1.06-1.47)	114/257 (44.4%)	1.80 (1.43-2.26)	.
Decrease	108/297 (36.4%)	0.74 (0.60-0.92)	812/1960 (41.4%)	1.22 (1.11-1.35)	249/598 (41.6%)	1.60 (1.38-1.86)	.

Supplementary Fig.S2. **Subgroup analysis for the associations between different patterns of BMI trajectories and the risk of hypertension in females.**

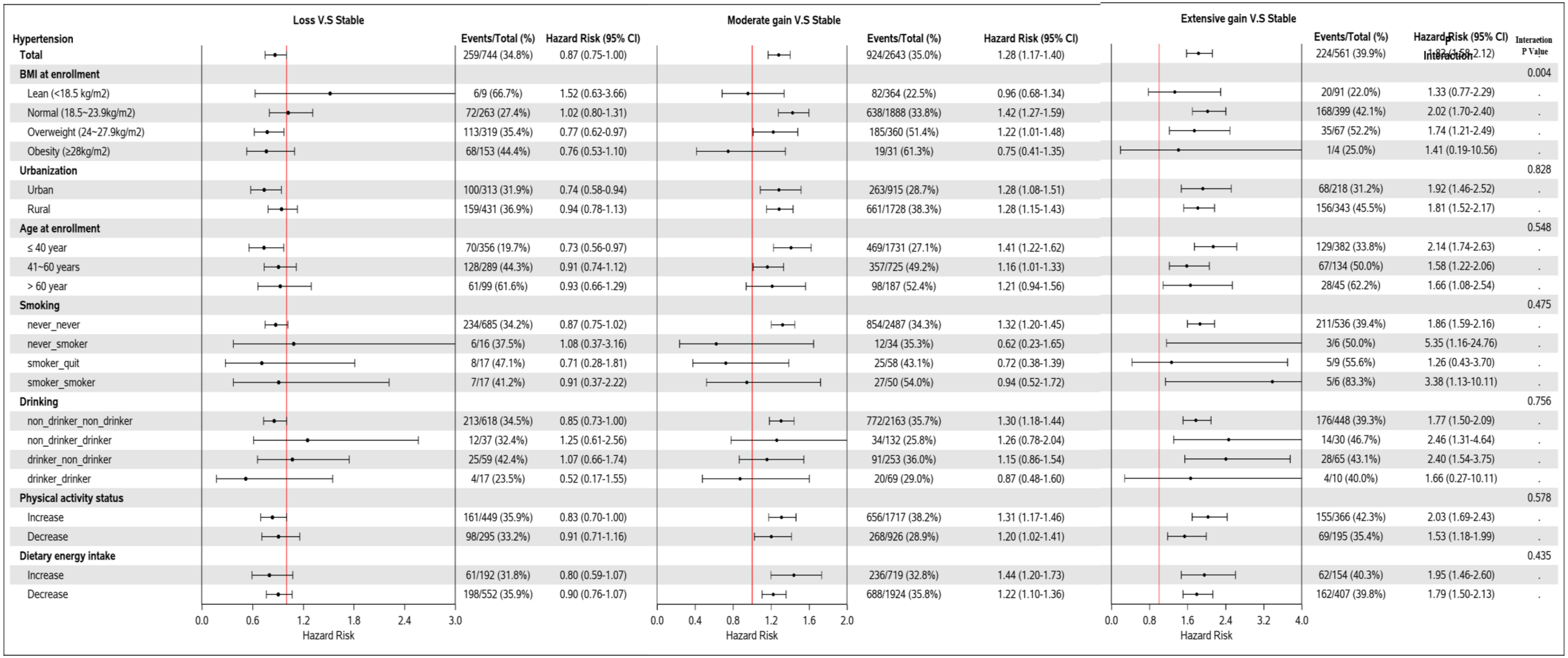

Hypertension	Loss V.S Stable: Events/Total (%)	Loss V.S Stable: Hazard Risk (95% CI)	Moderate gain V.S Stable: Events/Total (%)	Moderate gain V.S Stable: Hazard Risk (95% CI)	Extensive gain V.S Stable: Events/Total (%)	Extensive gain V.S Stable: Hazard Risk (95% CI)	Interaction P Value
**Total**	259/744 (34.8%)	0.87 (0.75-1.00)	924/2643 (35.0%)	1.28 (1.17-1.40)	224/561 (39.9%)	1.8[illegible] (1.58-2.12)	.
**BMI at enrollment**							0.004
Lean (<18.5 kg/m2)	6/9 (66.7%)	1.52 (0.63-3.66)	82/364 (22.5%)	0.96 (0.68-1.34)	20/91 (22.0%)	1.33 (0.77-2.29)	.
Normal (18.5~23.9kg/m2)	72/263 (27.4%)	1.02 (0.80-1.31)	638/1888 (33.8%)	1.42 (1.27-1.59)	168/399 (42.1%)	2.02 (1.70-2.40)	.
Overweight (24~27.9kg/m2)	113/319 (35.4%)	0.77 (0.62-0.97)	185/360 (51.4%)	1.22 (1.01-1.48)	35/67 (52.2%)	1.74 (1.21-2.49)	.
Obesity (≥28kg/m2)	68/153 (44.4%)	0.76 (0.53-1.10)	19/31 (61.3%)	0.75 (0.41-1.35)	1/4 (25.0%)	1.41 (0.19-10.56)	.
**Urbanization**							0.828
Urban	100/313 (31.9%)	0.74 (0.58-0.94)	263/915 (28.7%)	1.28 (1.08-1.51)	68/218 (31.2%)	1.92 (1.46-2.52)	.
Rural	159/431 (36.9%)	0.94 (0.78-1.13)	661/1728 (38.3%)	1.28 (1.15-1.43)	156/343 (45.5%)	1.81 (1.52-2.17)	.
**Age at enrollment**							0.548
≤ 40 year	70/356 (19.7%)	0.73 (0.56-0.97)	469/1731 (27.1%)	1.41 (1.22-1.62)	129/382 (33.8%)	2.14 (1.74-2.63)	.
41~60 years	128/289 (44.3%)	0.91 (0.74-1.12)	357/725 (49.2%)	1.16 (1.01-1.33)	67/134 (50.0%)	1.58 (1.22-2.06)	.
> 60 year	61/99 (61.6%)	0.93 (0.66-1.29)	98/187 (52.4%)	1.21 (0.94-1.56)	28/45 (62.2%)	1.66 (1.08-2.54)	.
**Smoking**							0.475
never_never	234/685 (34.2%)	0.87 (0.75-1.02)	854/2487 (34.3%)	1.32 (1.20-1.45)	211/536 (39.4%)	1.86 (1.59-2.16)	.
never_smoker	6/16 (37.5%)	1.08 (0.37-3.16)	12/34 (35.3%)	0.62 (0.23-1.65)	3/6 (50.0%)	5.35 (1.16-24.76)	.
smoker_quit	8/17 (47.1%)	0.71 (0.28-1.81)	25/58 (43.1%)	0.72 (0.38-1.39)	5/9 (55.6%)	1.26 (0.43-3.70)	.
smoker_smoker	7/17 (41.2%)	0.91 (0.37-2.22)	27/50 (54.0%)	0.94 (0.52-1.72)	5/6 (83.3%)	3.38 (1.13-10.11)	.
**Drinking**							0.756
non_drinker_non_drinker	213/618 (34.5%)	0.85 (0.73-1.00)	772/2163 (35.7%)	1.30 (1.18-1.44)	176/448 (39.3%)	1.77 (1.50-2.09)	.
non_drinker_drinker	12/37 (32.4%)	1.25 (0.61-2.56)	34/132 (25.8%)	1.26 (0.78-2.04)	14/30 (46.7%)	2.46 (1.31-4.64)	.
drinker_non_drinker	25/59 (42.4%)	1.07 (0.66-1.74)	91/253 (36.0%)	1.15 (0.86-1.54)	28/65 (43.1%)	2.40 (1.54-3.75)	.
drinker_drinker	4/17 (23.5%)	0.52 (0.17-1.55)	20/69 (29.0%)	0.87 (0.48-1.60)	4/10 (40.0%)	1.66 (0.27-10.11)	.
**Physical activity status**							0.578
Increase	161/449 (35.9%)	0.83 (0.70-1.00)	656/1717 (38.2%)	1.31 (1.17-1.46)	155/366 (42.3%)	2.03 (1.69-2.43)	.
Decrease	98/295 (33.2%)	0.91 (0.71-1.16)	268/926 (28.9%)	1.20 (1.02-1.41)	69/195 (35.4%)	1.53 (1.18-1.99)	.
**Dietary energy intake**							0.435
Increase	61/192 (31.8%)	0.80 (0.59-1.07)	236/719 (32.8%)	1.44 (1.20-1.73)	62/154 (40.3%)	1.95 (1.46-2.60)	.
Decrease	198/552 (35.9%)	0.90 (0.76-1.07)	688/1924 (35.8%)	1.22 (1.10-1.36)	162/407 (39.8%)	1.79 (1.50-2.13)	.

Supplementary Fig.S3. the directed acyclic graph among the potential covariates in the association between the pattern of longitudinal BMI change trajectories and hypertension

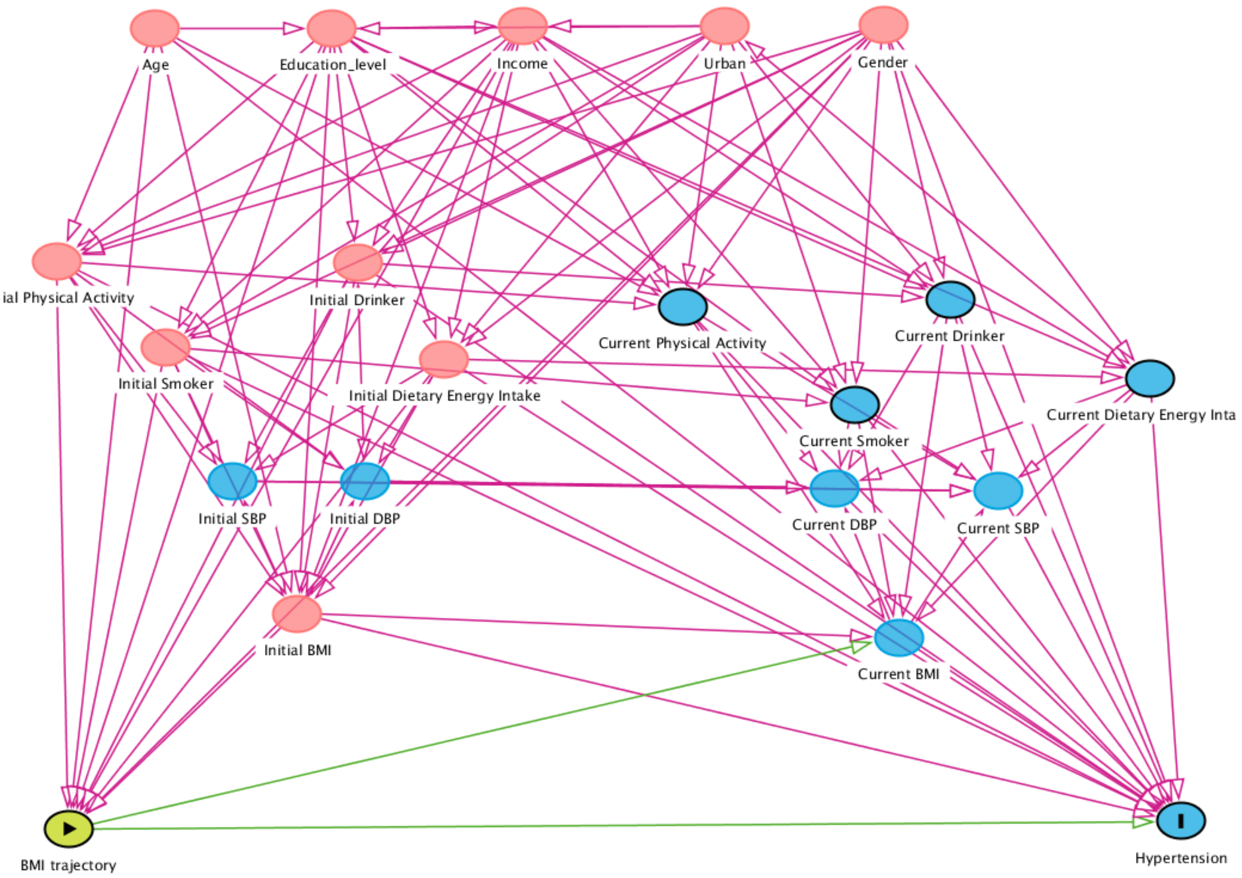

Supplement: Supplementary file 1 — Supplementary Information. [file 41598_2021_92319_MOESM1_ESM.pdf]
